# Supplementary material for: Distribution of Helicobacter pylori and Periodontopathic Bacterial Species in the Oral Cavity
Source: Biomedicines. 2020 Jun 15;8(6):161. doi: 10.3390/biomedicines8060161 (PMC7344611; doi:10.3390/biomedicines8060161)
Supplement: Supplementary file 1 [file biomedicines-08-00161-s001.pdf]

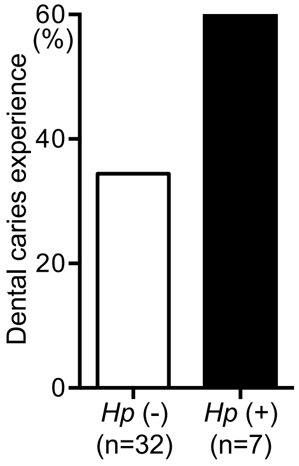

Supplementary figure 1. Rates of subjects with dental caries of the extracted teeth.

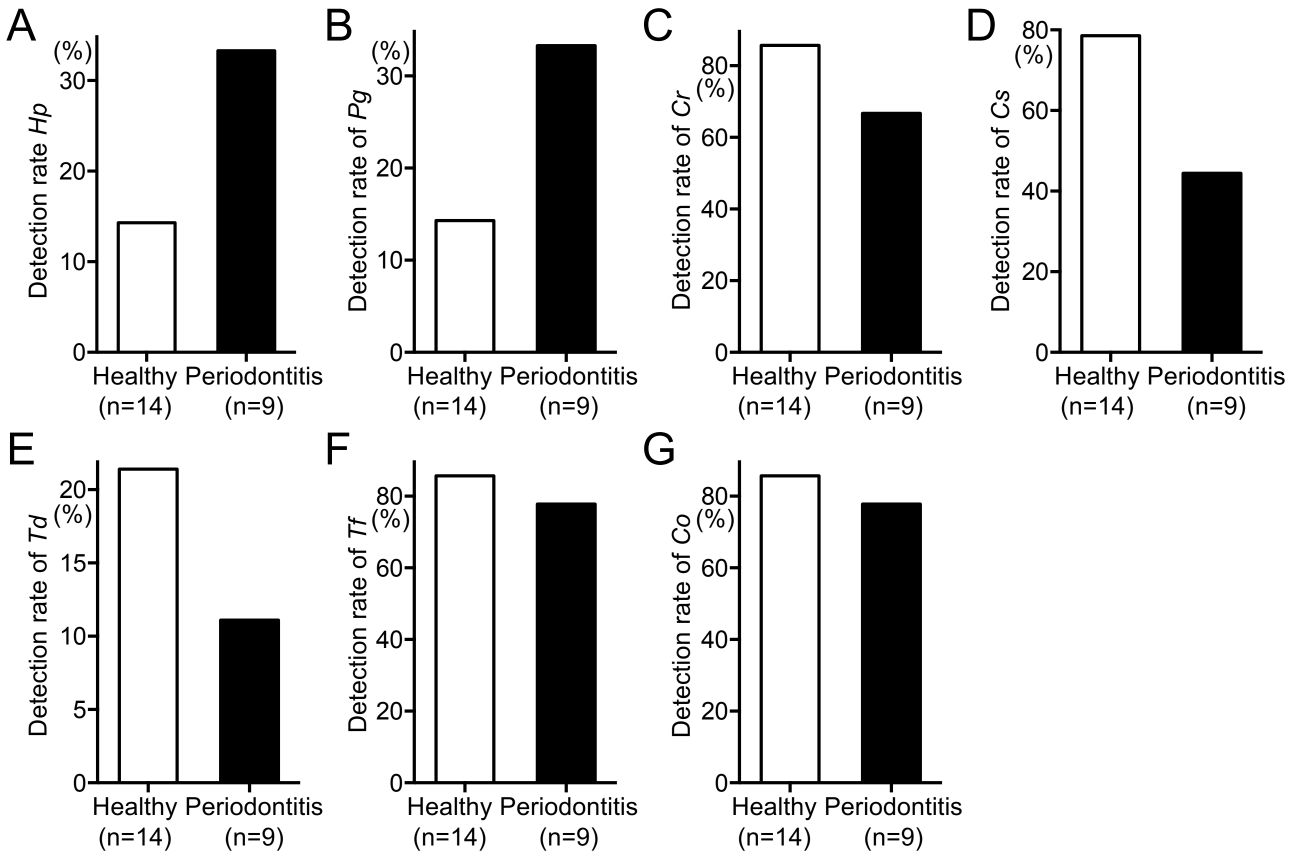

Supplementary figure 2. Rates of bacteria in dental plaques collected from healthy and periodontal teeth. Detection rates of (A) *H. pylori*, (B) *P. gingivalis*, (C) *C. rectus*, (D) *C. sputigena*, (E) *T. denticola*, (F) *T. forsythia*, and (G) *C. ochracea*. *Hp*, *H. pylori*; *Pg*, *P. gingivalis*; *Cr*, *C. rectus*; *Cs*, *C. sputigena*; *Td*, *T. denticola*; *Tf*, *T. forsythia*; and Co, *C. ochracea*.
